# Toward reproducible metabolic tumor volume quantification in endometrial cancer: optimizing [¹⁸F]FDG PET/CT tumor segmentation methods

**DOI:** 10.1186/s13550-026-01412-0

**Published:** 2026-05-28

**Authors:** Kristine Eldevik Fasmer, Sunniva Lindås, Ankush Gulati, Julie Andrea Dybvik, Erlend Hodneland, Camilla Krakstad, Ingfrid Salvesen Haldorsen

**Affiliations:** 1 Mohn Medical Imaging and Visualization Centre, Department of Radiology, Haukeland University Hospital, Bergen, Norway; 2 Department of Clinical Medicine, University of Bergen, Bergen, Norway; 3 Centre for Cancer Biomarkers, Department of Clinical Science, University of Bergen, Bergen, Norway; 4 Department of Obstetrics and Gynecology, Haukeland University Hospital, Bergen, Norway

**Keywords:** Endometrial cancer, Positron emission tomography computed tomography, Fluorodeoxyglucose F18, Interobserver agreement, Prognosis

## Abstract

**Background:**

Metabolic tumor volume (MTV) derived from [^18^F]FDG PET/CT has shown promise in improving preoperative staging and prognostication in endometrial cancer, both as a standalone biomarker and as a foundation for PET-based tumor radiomics. Despite its potential, standardized segmentation methods are lacking. This study aimed to (1) evaluate interobserver agreement in MTV delineation, (2) compare MTVs obtained through various thresholding approaches by examining their correlation with anatomical tumor volume (ATV) from MRI, and (3) assess their value for predicting aggressive disease.

**Results:**

This study included 146 patients with histologically confirmed endometrial cancer who underwent preoperative PET/CT imaging on a Siemens TruePoint (*n* = 67) or a Siemens Biograph Vision scanner (*n* = 79). MTV_2.5_ defined by a standardized uptake value (SUV) threshold larger than 2.5, demonstrated excellent reproducibility between readers, with an intraclass correlation coefficient (95% confidence interval (CI)) of 0.97 (0.96, 0.98). MTV_40%_, defined by SUV > 40% of SUV_max_, yielded an ICC (95% CI) of 0.91 (0.87, 0.93). Both the fixed 2.5 threshold and all relative SUV_max_-based thresholds (20–60% SUV_max_) produced MTVs with strong correlation to ATV, with Spearman’s rank correlation coefficients ranging from 0.806 to 0.874 (*p* < 0.001). The closest volumetric match to ATV was observed for MTV_30%_, with a median difference (95% CI) of -4% (-11%, 2%). In terms of predictive performance, MTV_2.5_ and MTV_20–50%_ yielded comparable results for predicting lymph node metastases, advanced FIGO stage (III-IV), and 3-year disease specific survival, with area under receiver operating characteristic curve (AUC) values (95% CI) ranging from 0.68 (0.53, 0.82) to 0.77 (0.65, 0.88). MTV_60%_ showed slightly lower prediction values across all outcomes, with AUCs (95% CI) between 0.64 (0.49, 0.80) and 0.72 (0.63, 0.82).

**Conclusion:**

MTV_2.5_ has excellent reproducibility and correlates well with anatomical volume. Both MTV_2.5_ and MTV_20–50%_ thresholds yield similar performances for predicting aggressive disease features, supporting their potential for clinical implementation in preoperative assessment of endometrial cancer.

**Supplementary Information:**

The online version contains supplementary material available at 10.1186/s13550-026-01412-0.

## Introduction

The global incidence of endometrial cancer is steadily increasing, particularly in medium- to high-income countries. In Norway, age-standardized incidence rates have risen from approximately 17 cases per 100,000 women in the 1970 s to a plateau of around 27 cases since the early 2000 s [1]. The rising trend is largely driven by increasing obesity rates and aging population—both well-established risk factors for endometrial cancer [2]. As the most common gynecologic malignancy in developed countries, endometrial cancer represents a growing public health challenge, with significant implications for healthcare resource allocation and long-term patient outcomes.

Despite advances in diagnostic imaging and therapeutic strategies, accurate preoperative risk stratification remains a clinical challenge in endometrial cancer. Current preoperative classification systems primarily rely on histopathological evaluation of endometrial biopsies, which broadly stratifies patients into low-risk (endometrioid endometrial carcinoma [EEC] grade 1–2) and high-risk (EEC grade 3 and non-endometrioid endometrial carcinoma [NEEC]) categories [2]. In addition, imaging modalities such as magnetic resonance imaging (MRI) and [^18^F]fluorodeoxyglucose ([^18^F]FDG) positron emission tomography (PET) combined with computed tomography (CT) are routinely employed to evaluate disease extent and inform surgical and adjuvant treatment decisions [3–7]. While pelvic MRI offers high specificity for assessing myometrial invasion and cervical stromal involvement [5, 7, 8], PET/CT provides superior sensitivity for detecting lymph node metastases (LNM) and distant spread [9, 10]. However, conventional imaging assessments are largely qualitative or based on simple anatomical measurements, which are unlikely to fully capture the biological aggressiveness of the tumor.

Among emerging quantitative imaging biomarkers, metabolic tumor volume (MTV) derived from [^18^F]FDG PET/CT has shown promise in enhancing preoperative staging and prognostication in endometrial cancer [9–11]. By quantifying the metabolically active portion of the tumor, MTV may provide a more comprehensive measure of tumor burden than conventional intensity-based PET tumor metrics such as maximum standardized uptake value (SUV_max_), or size-based MRI metrics like anatomical tumor volume (ATV) [10–12]. Moreover, accurate and reproducible MTV segmentation is a critical prerequisite for PET radiomics, an emerging image analysis technique that extracts high-dimensional tumor features for risk prediction and predictive modeling [13, 14].

However, the clinical utility of MTV—and radiomics more broadly—is constrained by the lack of standardized and reproducible tumor delineation protocols. The European Association of Nuclear Medicine (EANM) Guidelines for [^18^F]FDG PET/CT tumor imaging [15] recommend using a 41% SUV_max_ threshold for tumor delineation when radiotracer uptake is homogeneous and the tumor-to-background signal is high. In cases with inhomogeneous uptake, low tumor-to-background contrast, or image noise, higher thresholds—such as 50% SUV_max_—are advised to improve segmentation reliability [15].

The joint EANM/Society of Nuclear Medicine and Molecular Imaging (SNMMI) radiomics guideline [13] endorses SUV_max_-based thresholding methods due to their high inter-reader reproducibility. However, it underscores the importance of expert oversight and manual adjustments, particularly in cases with heterogeneous tumor uptake or variable background activity. The guideline further advocates for segmentation approaches that prioritize positive predictive value over sensitivity and recommends (semi)automated segmentation methods over manual delineation to minimize variability and improve consistency.

Notably, these guidelines are based on a limited number of studies, primarily involving lung, esophageal, and breast cancers, and also, phantom and simulation models. Consequently, their generalizability to other tumor types—such as endometrial tumors located near metabolically active organs like the bladder—remains uncertain and warrants further investigation.

To date, no studies have systematically evaluated optimal thresholding methods for MTV quantification in endometrial cancer, limiting its integration into clinical practice. This study aims to identify an optimal MTV segmentation approach by evaluating interobserver agreement and examining correlations between MTV and MRI-derived ATV. Additionally, we aim to evaluate and compare the predictive performances of the different MTV thresholds, for identifying aggressive disease features and poor outcome in endometrial cancer. By addressing the methodological variability in MTV quantification, this study seeks to advance the potential integration of metabolic imaging biomarkers into routine clinical workflows in endometrial cancer management.

## Materials and methods

### Patient cohort

This retrospective study utilized data from a prospectively maintained, population-based cohort of patients diagnosed with endometrial cancer and treated at Haukeland University Hospital (HUH) in Bergen, Norway. As part of the routine diagnostic workup, preoperative pelvic MRI and [^18^F]FDG PET/CT have been performed since 2009 and 2011, respectively [16]. The study was approved by the Regional Committee for Medical and Health Research Ethics (REK Vest, reference number 2015/2333), and written informed consent was obtained from all participants at the time of primary diagnosis.

**Fig. 1:**
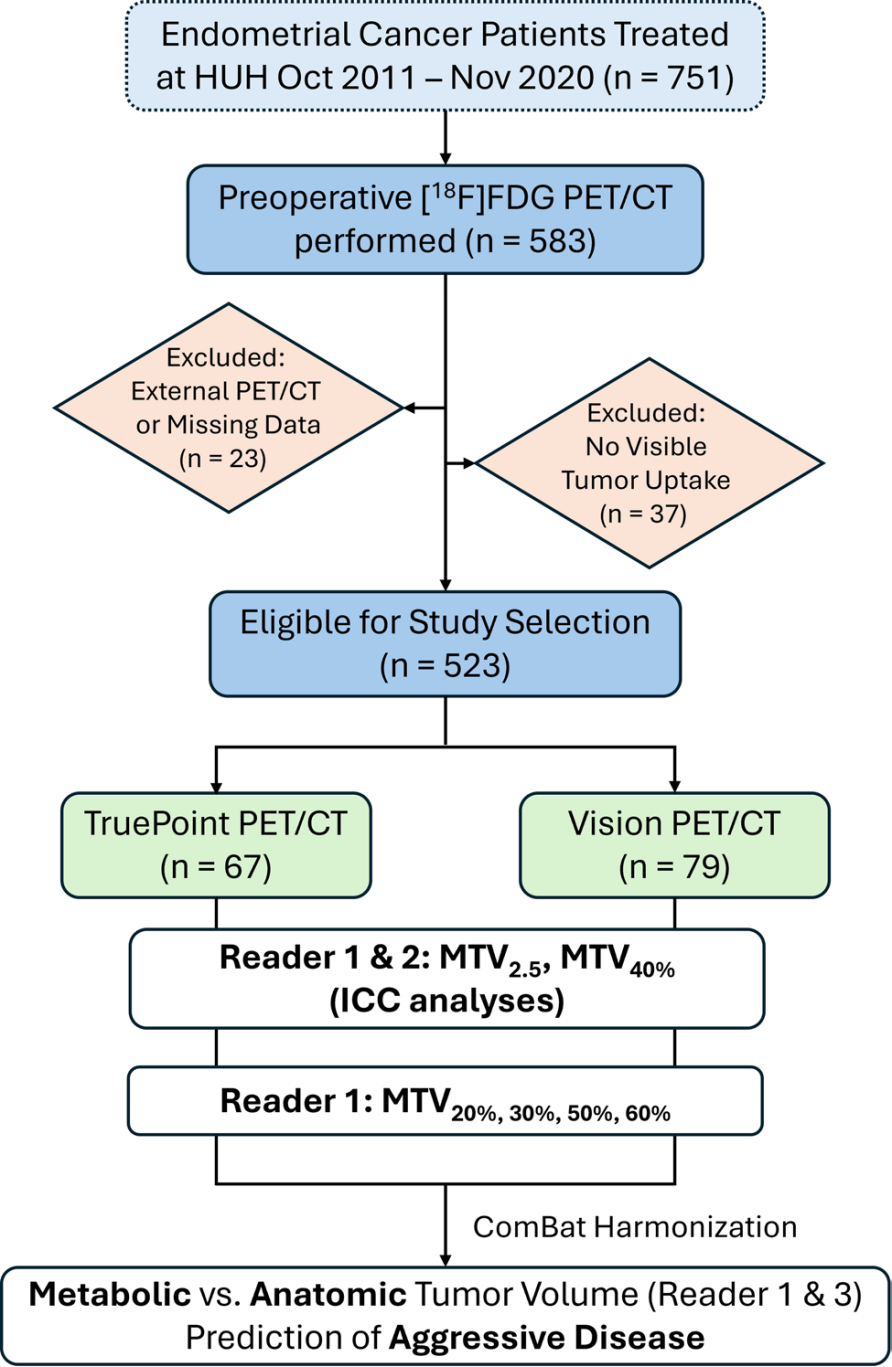
**Study flowchart**. Between October 2011 and November 2020, a total of 751 patients were treated for endometrial cancer at Haukeland University Hospital (HUH, Bergen, Norway). Of these, 583 patients underwent preoperative [^18^F]FDG PET/CT. Sixty patients (60/583) were excluded due to missing image data, PET/CT performed at external institutions, or absence of visible tumor uptake. From the remaining cohort, 146 patients were randomly selected, 67 patients imaged using Siemens TruePoint PET/CT (2011- late 2018) and 79 patients using Siemens Vision PET/CT (late 2018–2020). Metabolic tumor volumes (MTVs) were delineated independently by two readers (Reader 1 and 2) using thresholds of SUV > 2.5 (MTV_2.5_) and SUV > 40% SUV_max_ (MTV_40%_) to assess interobserver agreement via intraclass correlation coefficients (ICCs). Reader 1 additionally generated four SUV_max_-based MTVs (MTV_20%_, MTV_30%_, MTV_50%_, MTV_60%_). All MTVs derived by Reader 1 were compared with anatomical tumor volume from contrast-enhanced T1-weighted MRI (Reader 3) and evaluated for prediction of aggressive disease and adverse clinical outcomes. Abbreviations: [^18^F]FDG, fluorodeoxyglucose; MRI, magnetic resonance imaging; PET/CT, positron emission tomography combined with computed tomography; SUV, standardized uptake value

Between October 2011 and November 2020, a total of 751 patients were treated for endometrial cancer at HUH. Of these, 583 patients underwent preoperative [^18^F]FDG PET/CT. Sixty patients (60/583) were excluded due to missing image data, PET/CT performed at external institutions, or absence of visible tumor uptake. From the remaining eligible cohort, 146 patients were randomly selected for inclusion in this study, however aiming to achieve a balanced distribution across PET/CT scanner types: Siemens Biograph Vision (*n* = 79; late 2018–2020) and Siemens Biograph TruePoint (*n* = 67; 2011– late 2018) (Fig. 1).

### PET/CT and MRI protocols

All PET/CT examinations were conducted in accordance with the EANM guidelines [15]. Patients fasted for a minimum of six hours prior to the scanning, and blood glucose levels were controlled before intravenous administration of [^18^F]FDG. Following tracer injection, patients rested in a quiet room for approximately 60 min to allow for adequate tracer uptake.

PET images were acquired from the skull base to mid-thigh and corrected for attenuation and scatter using the corresponding CT images. Two PET/CT systems were used during the study period: Siemens Biograph TruePoint (from 2011 to late 2018) and Siemens Biograph Vision (from late 2018 to 2020). Acquisition and reconstruction parameters were tailored to each scanner in accordance with institutional protocols; detailed specifications are provided in Supplementary Table S1.

Preoperative pelvic MRI was performed on a 1.5 T Siemens Avanto, or on a 3 T Siemens Skyra scanner. The imaging sequence used to segment ATVs was a contrast-enhanced, T1-weighted axial oblique 3D volumetric interpolated breath-hold (VIBE) gradient echo sequence with fat saturation, acquired two minutes after contrast administration (Supplementary Table S1).

### PET/CT metabolic tumor segmentation and extraction of tumor metrics

All PET/CT images were independently evaluated by two readers. Reader 1 (R1) was a medical student at the University of Bergen with no prior experience in PET/CT interpretation at the start of the project. Reader 2 (R2) is a board-certified Nuclear Medicine Physician at Haukeland University Hospital with more than seven years of experience in PET/CT interpretation. Prior to image analyses, R1 received dedicated training and supervision from R2.

**Fig. 2:**
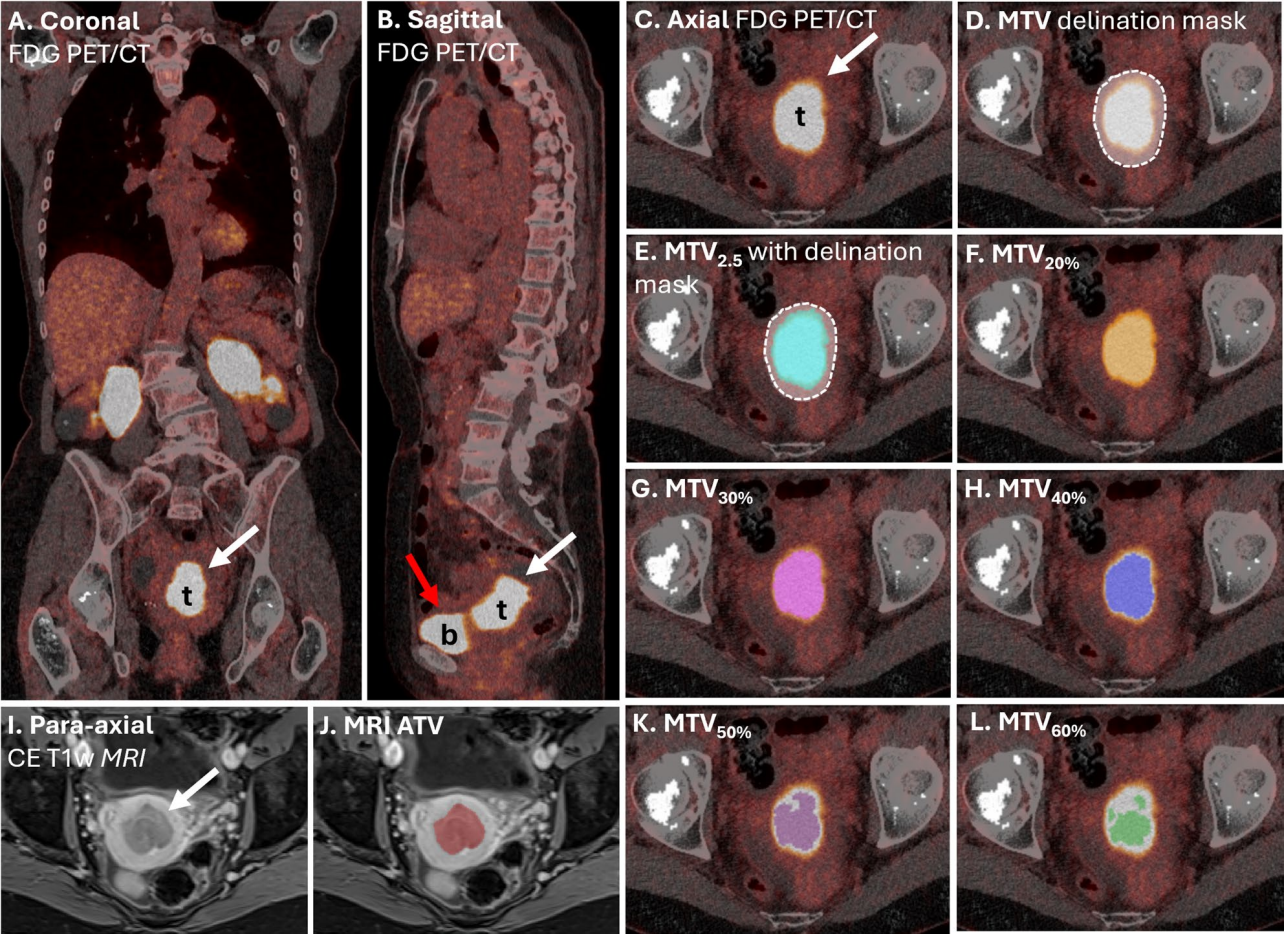
**Metabolic and anatomical tumor volume segmentation**. [^18^F]FDG PET/CT images in coronal (**A**), sagittal (**B**), and axial (**C**) views depict a primary endometrial tumor (t, white arrows) in a patient diagnosed with grade 2 endometrioid endometrial carcinoma, FIGO (2009) stage 3C1. The outer tumor mask (in gray with dotted line, **D**-**E**) was manually delineated slice-by-slice to encompass the tumor while excluding adjacent physiological uptake, such as from the bladder (b, red arrow). Within this mask, different standardized uptake value (SUV) thresholds were applied to define metabolic tumor volumes (MTVs): SUV > 2.5 (**E**), and relative thresholds of SUV > 20% (**F**), 30% (**G**), 40% (**H**), 50% (**K**), and 60% (**L**) of SUV_max_. Additionally, para-axial contrast enhanced (CE) T1-weighted MRI shows the primary tumor (white arrow, I), with the segmented anatomical tumor volume (ATV) highlighted in red (**J**). Abbreviations: [^18^F]FDG, fluorodeoxyglucose; FIGO, the International Federation of Gynecology and Obstetrics staging system; MRI, magnetic resonance imaging; PET/CT, positron emission tomography combined with computed tomography

The MTV segmentations were performed using the freely available LIFEx software (version 7.4.0; https://www.lifexsoft.org) [28]. Outer tumor boundaries were independently and manually delineated slice-by-slice by both readers to encompass all visible tumor tissue (Fig. 2). Following these manual segmentations, various standardized uptake value (SUV) thresholds were applied to generate MTVs.

R1 applied six SUV thresholds: a fixed threshold of SUV > 2.5 (MTV_2.5_), and five relative SUV_max_-based thresholds of SUV > 20%, 30%, 40%, 50% and 60% of SUV_max_ (MTV_20%_–MTV_60%_) (Fig. 2). For each MTV, the following PET-based tumor metrics were derived: SUV_max_, SUV_90p_, SUV_mean_¸ SUV_median_, and total lesion glycolysis (TLG), calculated as MTV × SUV_mean_.

R2 independently applied the fixed threshold of SUV > 2.5 (MTV_2.5_) and one relative threshold of SUV > 40% of SUV_max_ (MTV_40%_). Corresponding PET-based tumor metrics (SUV_max_, SUV_90p_, SUV_mean_¸ SUV_median_, and TLG) were subsequently extracted.

### MRI anatomical tumor volume segmentation

MRI-based ATVs were segmented by a radiology resident with more than four years of experience in interpreting pelvic MRI (Reader 3). Tumor delineation was conducted either manually, on a slice-by-slice basis, or by reviewing and adjusting segmentations generated by a previously validated machine learning model [20] (Fig. 2). The ATVs have been used in prior studies published by our research group [20–22]. In the present study, they serve exclusively as a reference for comparing anatomical and metabolic tumor volumes.

### Statistical analyses

All statistical analyses were performed in STATA version 18.0 (StataCorp LLC, College Station, TX, USA) and R version 4.5.2 (https://www.r-project.org/). 

Comparisons of clinicopathological characteristics between patients scanned on different PET/CT systems were performed using the Mann–Whitney exact U-test for continuous variables and Fisher’s exact test for categorical variables.

Interobserver agreement for PET-derived tumor metrics between Reader 1 and Reader 2 was assessed using intraclass correlation coefficients (ICCs), calculated with a two-way random-effects model for individual absolute agreement.

To account for scanner-related variability in PET metrics, post hoc ComBat harmonization [23] was applied using an online tool (https://forlhac.shinyapps.io/Shiny_ComBat/). ComBat is an empirical Bayes method that removes scanner- or protocol-specific shifts in mean and variance, while preserving true biological variation. In the context of PET imaging, ComBat harmonizes SUV-based metrics without the need for phantom measurements and enables more reliable pooling of quantitative data obtained across different PET systems/protocols. In this study, all PET-derived metrics were ComBat harmonized prior to analyses involving MTV-ATV-comparisons and prediction of clinical outcomes.

The concordance between MTVs derived by Reader 1 and ATVs derived by Reader 3 was evaluated using Wilcoxon’s matched-pair signed rank test and Spearman’s rank correlation coefficients (ρ). Bland–Altman plots were generated to visualize the mean differences between MTVs derived from various SUV thresholds and the corresponding ATVs.

The performance of the different MTVs (Reader 1) for prediction of LNM and FIGO stage III–IV was assessed using receiver operating characteristic (ROC) curve analysis implemented in the “pROC” package in R. Time-dependent ROC (tdROC) curves generated with the “timeROC” package were used to evaluate the performance of the MTVs for predicting 3-year DSS.

p-values of less than 0.05 were considered to represent statistically significant findings.

## Results

### Clinical findings, histopathology, and patient survival

In the presented endometrial cancer cohort, the median (interquartile range [IQR]) patient age at diagnosis was 69 (61–76) years, and 98% (143/146) of the patients underwent hysterectomy as primary treatment (Table 1). Lymphadenectomy was performed in 60% (88/146) of the patients and LNM were surgicopathologically confirmed in 9% (14/146) of the patients. After surgicopathological assessments, 86% (125/146) of patients were staged as FIGO (2009) stage I–II, and 14% (21/146) were staged as FIGO stage III–IV. Additional treatment was given to 43% (62/146) of the patients; mainly chemotherapy (54/62 patients). Median (IQR) [range] follow–up time for survivors was 46 (36–70) [3–98] months. The clinicopathological patient characteristics were overall similar across the two scanner systems used (*p* ≥ 0.05 for all, Table 1).

**Table 1:** Clinicopathological characteristics of the study cohort

	All patients (*n* = 146)	TruePoint (*n* = 67)	Vision (*n* = 79)	p*
Age(years), median (IQR)	69 (61–76)	69 (60–76)	69 (62–76)	0.87
Preoperative histology, n (%)				0.16
low-risk (EEC grade 1–2)	91 (62)	46 (69)	45 (57)	
high-risk (EEC grade 3/NEEC)	52 (36)	21 (31)	31 (39)	
missing	3 (2)	-	3 (4)	
Primary treatment, n (%)				0.16
hysterectomy	143 (98)	67 (100)	76 (96)	
curettage/palliative surgery	3 (2)	-	3 (4)	
Lymphadenectomy, n (%)				0.60
pelvic	51 (35)	26 (39)	25 (32)	
pelvic and paraaortic	37 (25)	15 (22)	22 (28)	
no	58 (40)	26 (39)	32 (40)	
Lymph node metastasis, n (%)				0.12
yes	14 (9)	9 (13)	5 (6)	
no	74 (51)	32 (48)	42 (53)	
not assessed	58 (40)	26 (39)	32 (41)	
Postoperative histology, n (%)				0.05
EEC grade 1–2	89 (61)	45 (67)	44 (56)	
EEC grade 3/NEEC	54 (37)	19 (28)	35 (44)	
missing	3 (2)	3 (5)	-	
FIGO stage, n (%)				0.71
I	113 (78)	52 (78)	61 (78)	
II	12 (8)	4 (6)	8 (10)	
III	18 (12)	9 (13)	9 (11)	
IV	3 (2)	2 (3)	1 (1)	
Additional treatment, n (%)				0.09
no	84 (57)	43 (64)	41 (52)	
yes	62 (43)	24 (36)	38 (48)	
chemotherapy	54 (37)	22 (33)	32 (40)	
radiotherapy (internal/external)	3 (2)	1 (1.5)	2 (3)	
chemoradiotherapy	2 (1)	0 (0)	2 (3)	
hormonal treatment	1 (1)	0 (0)	1 (1)	
other	2 (1)	1 (1.5)	1 (1)	

Clinical and pathological patients characteristics are presented for the entire study cohort (n = 146) and stratified by PET/CT scanner type: Siemens TruePoint PET/CT (n = 67) and Siemens Vision PET/CT (n = 79)

EEC, endometrioid endometrial carcinoma; FIGO, International Federation of Gynecology and Obstetrics; IQR, interquartile range; NEEC, non-endometrioid endometrial carcinoma; PET/CT, positron emission tomography combined with computed tomography

*Mann-Whitney U-test for continuous variables (exact p), and Fisher’s exact test for categorical variables, comparing patients on Siemens TruePoint vs. Siemens Vision. *p* < 0.05 marked in *italics*

### Metabolic tumor volume interobserver agreement

Table 2 presents median MTV_2.5_ and MTV_40%_ values together with the associated SUV metrics independently assessed by Reader 1 and 2, along with the corresponding ICCs. For MTV_2.5_, interobserver agreement was excellent for all evaluated metrics, including MTV_2.5_, SUV_max_, SUV_90p_, SUV_mean_, SUV_median_, and TLG across the entire cohort (ICC ≥ 0.90 for all). The highest agreement was observed for SUV_max_ (ICC [95% CI] = 0.99 [0.98–0.99]) and MTV_2.5_ (ICC [95% CI] = 0.97 [0.96–0.98]). Subgroup analysis by PET/CT scanner type revealed consistently higher interobserver agreement for patients scanned on the newer Siemens Vision system (ICC range: 0.93–1.00) compared with those scanned on the older Siemens TruePoint system (ICC range: 0.76–0.97).

**Table 2:** Metabolic tumor volume interobserver agreement

	Reader 1 median (95% CI)		Reader 2 median (95% CI)		ICC* (95% CI) All patients (*n* = 167)	ICC* (95% CI) TruePoint (*n* = 67)		ICC* (95% CI) Vision (*n* = 79)
MTV_2.5_ (mL)	20.5 (16.2, 24.0)		19.7 (16.3, 24.0)		*0.97 (0.96, 0.98)*	*0.97 (0.95, 0.98)*		*0.98 (0.96, 0.99)*
SUV_max_	17.0 (15.5, 18.3)		17.0 (15.3, 18.3)		*0.99 (0.98*,* 0.99)*	*0.94 (0.91*,* 0.97)*		*1.00 (1.00*,* 1.00)*
SUV_90p_	11.0 (10.0, 11.9)		11.0 (10.1, 11.9)		*0.95 (0.93*,* 0.96)*	*0.83 (0.74*,* 0.89)*		*1.00 (0.99*,* 1.00)*
SUV_mean_	6.0 (5.7, 6.5)		6.2 (5.6, 6.7)		*0.94 (0.92*,* 0.96)*	*0.82 (0.72*,* 0.88)*		*0.98 (0.96*,* 0.99)*
SUV_median_	5.0 (4.7, 5.4)		5.2 (4.7, 5.6)		*0.90 (0.86*,* 0.93)*	*0.76 (0.63*,* 0.84)*		*0.93 (0.89*,* 0.95)*
TLG (mL)	141 (99, 172)		130 (97, 161)		*0.94 (0.91*,* 0.95)*	*0.87 (0.80*,* 0.92)*		*0.99 (0.99*,* 1.00)*
MTV_40%_ (mL)	7.1 (5.7, 8.6)		7.0 (5.9, 8.7)		*0.91 (0.87*,* 0.93)*	*0.90 (0.83*,* 0.93)*		*0.93 (0.90*,* 0.96)*
SUV_max_	17.0 (15.3, 18.3)		16.7 (15.3, 18.2)		*0.93 (0.90*,* 0.95)*	*0.80 (0.70*,* 0.87)*		*0.98 (0.96*,* 0.99)*
SUV_90p_	13.1 (12.4, 14.4)		13.1 (12.2, 14.4)		*0.91 (0.88*,* 0.93)*	*0.79 (0.68*,* 0.86)*		*0.97 (0.95*,* 0.98)*
SUV_mean_	10.2 (9.1, 10.8)		10.1 (8.9, 10.7)		*0.89 (0.85*,* 0.92)*	*0.75 (0.63*,* 0.84)*		*0.95 (0.92*,* 0.97)*
SUV_median_	9.7 (8.7, 10.3)		9.7 (8.6, 10.3)		*0.87 (0.82*,* 0.90)*	*0.73 (0.59*,* 0.82)*		*0.93 (0.90*,* 0.96)*
TLG (mL)	72 (58, 97)		66 (56, 96)		*0.78 (0.71*,* 0.84)*	*0.56 (0.37*,* 0.70)*		*0.98 (0.97*,* 0.99)*

Median (95% CI) [^18^F]FDG PET-derived metrics for primary endometrial tumors, assessed by two independent readers (R1, R2) using metabolic tumor volume thresholds of SUV > 2.5 (MTV_2.5_) and SUV > 40% of SUV_max_ (MTV_40%_). ICCs (95% CI) are reported for the entire patient cohort (n = 146), and separately for patients scanned on Siemens TruePoint (n = 67) and Siemens Vision (n = 79)

CI, confidence interval; [^18^F]FDG, fluorodeoxyglucose; ICC, intraclass correlation coefficient; PET, positron emission tomography; SUV, standardized uptake value; TLG, tumor lesion glycolysis; 90p, 90 percentile

*ICC individual absolute agreement. ICCs with *p* < 0.001 marked in *italics*

For MTV_40%_, interobserver agreement was also high (ICCs ≥ 0.78 for the entire cohort), although slightly lower than for MTV_2.5_. Agreement was highest for SUV_max_ (ICC [95% CI] = 0.93 [0.90–0.95]), and lowest for TLG (ICC [95% CI] = 0.78 [0.71–0.84]). As with MTV_2.5_, ICCs were higher for scans acquired on the Siemens Vision system (ICC range: 0.93–0.98) compared with the Siemens TruePoint system (ICC range: 0.56–0.90).

### Harmonization of PET tumor metrics derived on two scanner systems

Significant differences were observed in the distribution of several PET-derived tumor metrics between the two scanner systems, independent of the MTV threshold applied (see Supplementary Table S2). To address this variability, ComBat harmonization was applied, effectively eliminating inter-scanner discrepancies (Supplementary Table S2). Since clinicopathological characteristics were comparable across the scanner systems (Table 1), no covariates were included in the harmonization process. All subsequent analyses involving pooled data from the two systems were conducted using the harmonized PET metrics (from Reader 1) to ensure consistency and comparability across the dataset.

### Comparison of anatomical and metabolic tumor volumes

**Fig. 3:**
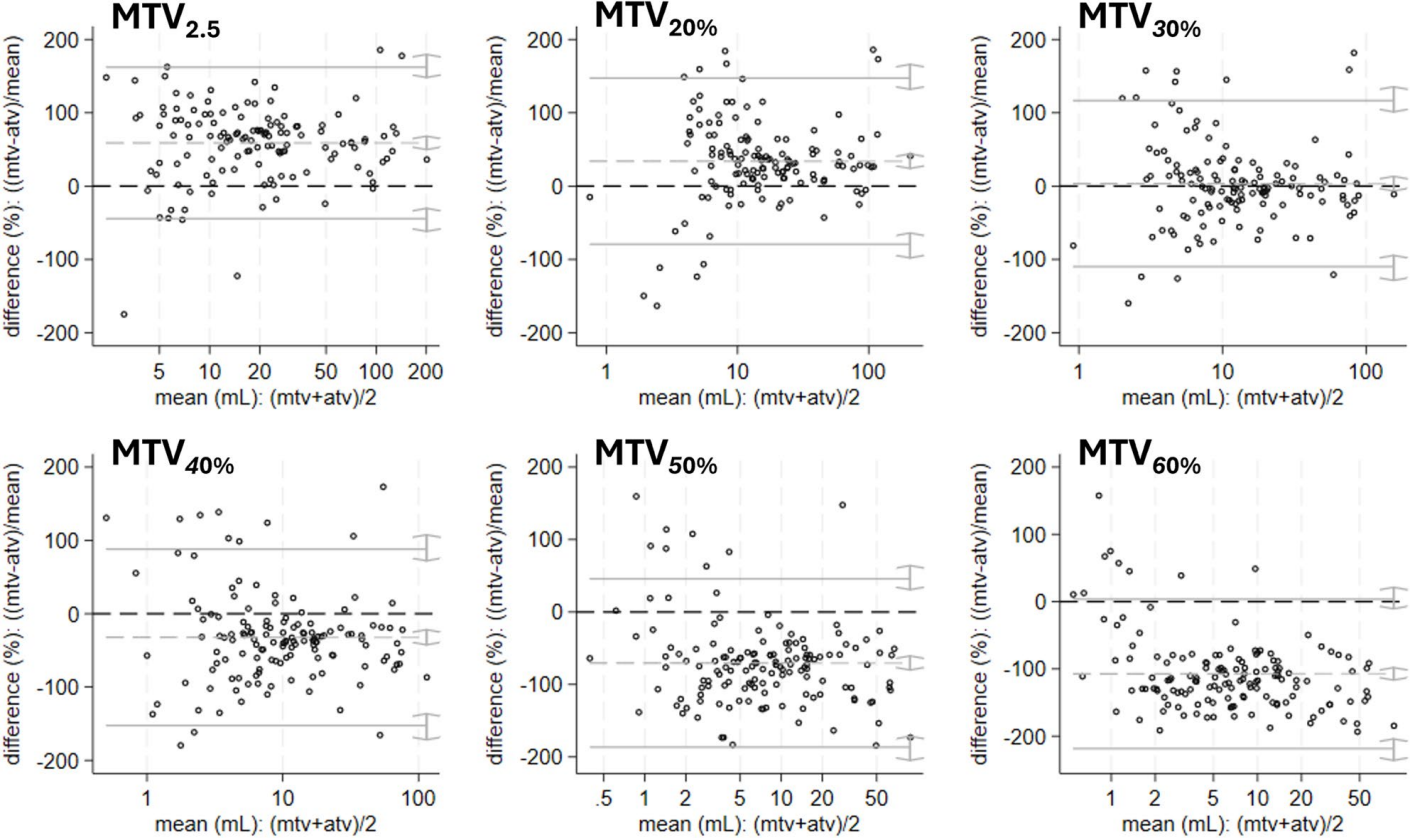
**Comparison of anatomical and metabolic tumor volumes**. Bland-Altman plots depicting differences (%) between MRI-derived anatomical tumor volume (ATV, Reader 3), and [^18^F]FDG PET-derived ComBat harmonized metabolic tumor volumes (MTV_2.5_ and MTV_20−60%_, Reader 1) in 146 endometrial cancer patients. Horizontal lines depict mean difference (dotted lines) with 95% confidence intervals (continuous lines). Abbreviations: [^18^F]FDG, fluorodeoxyglucose; MRI, magnetic resonance imaging; PET, positron emission tomography

The median (95% CI) ATV was 9.5 (8.0–12.3) mL, while MTVs varied substantially depending on the threshold applied (Table 3; Fig. 3). MTV_2.5_ yielded the largest median volume (20.4 mL, 95% CI: 15.5–24.4), with a median difference of 85% (95% CI: 63–105%) compared to ATV. In contrast, MTV_60%_ produced the smallest median volume (2.4 mL, 95% CI: 1.8–3.2), corresponding to a − 73% difference to ATV (95% CI: −78 to − 71%). The best volumetric agreement with ATV was observed for MTV_30%_ (median difference: −4%, 95% CI: −11 to 2%) (Fig. 3).

**Table 3:** Comparison of anatomical and metabolic tumor volumes

	Median volume (mL) (95% CI)	Median difference (%) (95% CI)*	Correlation coefficient (ρ)**
ATV	9.5 (8.0, 12.3)	na	na
MTV_2.5_	20.4 (15.5, 24.4)	*85% (63, 105%)*	*0.874*
MTV_20%_	14.3 (12.6, 17.4)	*32% (23, 44%)*	*0.870*
MTV_30%_	9.9 (8, 12.1)	-4% (-11, 2%)	*0.867*
MTV_40%_	7.0 (5.3, 8.8)	*-31% (-36, -27%)*	*0.864*
MTV_50%_	4.1 (3.4, 5.8)	*-53% (-57, -49%)*	*0.858*
MTV_60%_	2.4 (1.8, 3.2)	*-73% (-78, -71%)*	*0.806*

Median (95% CI) anatomical tumor volume (ATV) derived from MRI (Reader 3), and median (95% CI) ComBat harmonized metabolic tumor volumes (MTV) derived from [^18^F]FDG PET (Reader 1) using six different thresholds: SUV > 2.5 and SUV > 20%-60% of SUV_max_. The different MTVs are compared with the ATV by median (95% CI) differences (%) and Spearman correlation coefficients (ρ)

CI, confidence interval; [^18^F]FDG, fluorodeoxyglucose; MRI, magnetic resonance imaging; na, not applicable; PET, positron emission tomography; SUV, standardized uptake value

*Wilcoxon matched-pair signed-rank test comparing *MTV* and *ATV. *p<0.001 marked in *italics*

**Spearman’s rank correlation coefficient, rho (ρ). Coefficients with p<0.001 marked in *italics*

All MTV thresholds demonstrated strong positive correlations with ATV, with Spearman’s ρ ranging from 0.806 (MTV_60%_) to 0.874 (MTV_2.5_), all statistically significant (*p* < 0.001 for all; Table 3).

### PET metrics for predicting aggressive disease and poor survival

**Fig. 4:**
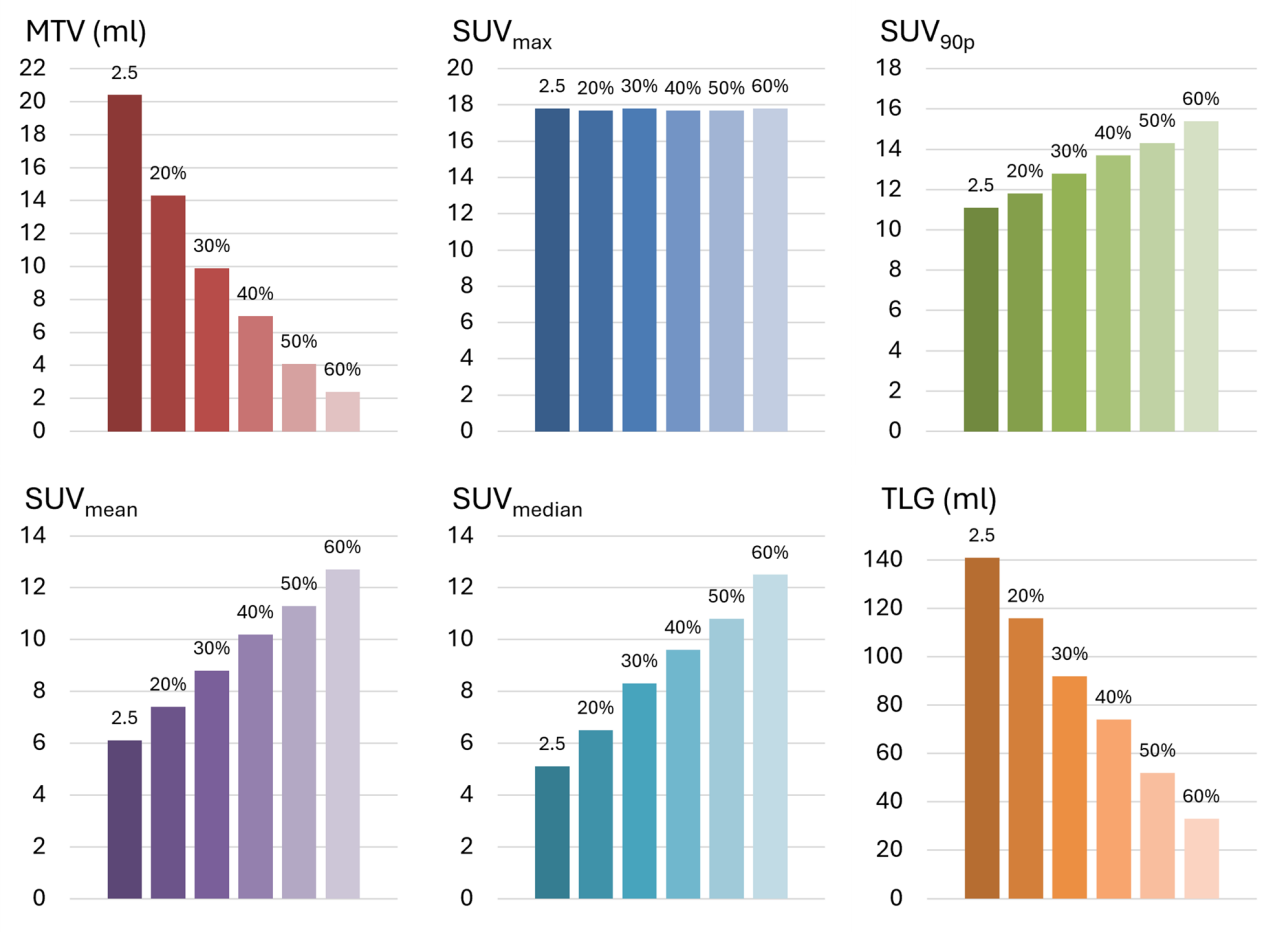
**PET metrics derived using different tumor thresholds**. Median ComBat harmonized [^18^F]FDG PET-derived metrics for primary endometrial tumors, assessed by Reader 1 using different metabolic tumor volume (MTV) thresholds: SUV > 2.5 and SUV > 20%−60% of SUV_max_ Abbreviations: [^18^F]FDG, fluorodeoxyglucose; PET, positron emission tomography; SUV, standardized uptake value; TLG, tumor lesion glycolysis

Figure 4 illustrates the variation in median PET-derived tumor metrics—MTV, SUV_max_, SUV_90p_, SUV_mean_, SUV_median_ and TLG—across the six different MTV thresholds applied. While SUV_max_ remained stable across all thresholds, SUV_90p_, SUV_mean_, and SUV_median_ showed a gradual increase with higher thresholds, reflecting the increasingly selective inclusion of only the most metabolically active tumor regions. In contrast, TLG decreased with increasing thresholds, consistent with the corresponding reduction in MTV.

**Fig. 5:**
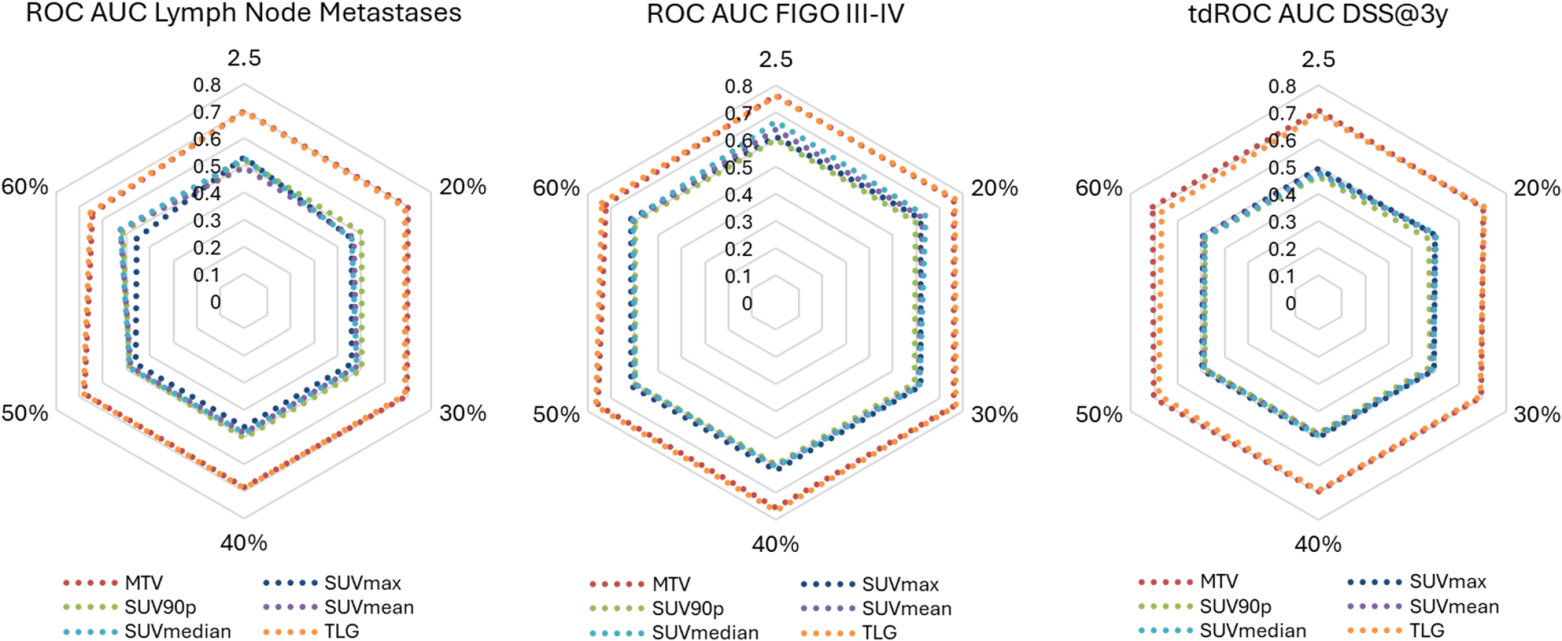
**PET metrics for predicting aggressive disease and disease specific survival**. Radar plot depicting predictive performances for the ComBat harmonized [^18^F]FDG PET metrics derived by Reader 1 using different metabolic tumor volume (MTV) threshold (SUV > 2.5 and SUV > 20%−60% of SUV_max_). Performances were evaluated using areas under receiver operating characteristics curves (AUC ROCs) for predicting lymph node metastases (LNM) and advanced stage (FIGO III-IV), and time-dependent (td) AUC tdROCs for predicting 3-year disease specific survival (DSS). Abbreviations: [^18^F]FDG, fluorodeoxyglucose; FIGO, International Federation of Gynecology and Obstetrics; PET, positron emission tomography; SUV, standardized uptake value; TLG, tumor lesion glycolysis

For prediction of the clinical endpoints: LNM, FIGO stage III-IV and 3-year DSS, MTV and TLG consistently demonstrated the highest discriminative performance with AUCs (95% CI) ranging from 0.64 (0.49, 0.80) to 0.70 (0.54, 0.86) for LNM; 0.72 (0.63, 0.82) to 0.77 (0.67, 0.87) for FIGO stage III-IV, and 0.67 (0.53, 0.81) to 0.71 (0.54, 0.87) for 3-year DSS (Fig. 5, Supplementary Table S3). In comparison, SUV_max_, SUV_90p_, SUV_mean_, and SUV_median_ showed lower AUCs (95% CI) across all endpoints: AUCs ≤ 0.53 (0.36, 0.70) for LNM; ≤ 0.67 (0.56, 0.77) for FIGO III-IV; and ≤ 0.50 (0.35, 0.66) for DSS.

Although differences in predictive performance across MTV thresholds were generally modest, there was a trend toward reduced performance for predicting LNM and FIGO stage III–IV when using the highest threshold (60% of SUV_max_), suggesting that high segmentation thresholds may reduce the MTV’s prognostic utility (Fig. 5, Supplementary Table S3).

## Discussion

In this study, we evaluated the reproducibility and clinical utility of various MTV segmentation methods derived from pretreatment [^18^F]FDG PET/CT in a well-characterized cohort of endometrial cancer patients. Our findings demonstrate that the fixed SUV threshold method (MTV_2.5_) yielded excellent interobserver agreement, while relative SUV_max_-based thresholds—particularly MTV_30%_—most closely approximated ATV derived from MRI. All MTV thresholds showed strong correlations with ATV, and MTV consistently outperformed intensity-based PET metrics (e.g., SUV_max_) in predicting lymph node metastases, advanced FIGO stage, and 3-year disease-specific survival. These results support the role of MTV as a reproducible and clinically informative imaging biomarker in endometrial cancer and highlight the importance of selecting MTV delineation thresholds that balance reproducibility, anatomical accuracy, and prognostic value.

Accurate preoperative risk stratification is essential for guiding surgical and adjuvant treatment decisions in endometrial cancer. The FIGO 2023 staging system [17] places increased emphasis on prognostic factors, such as histological type, tumor grade, molecular classification, and lymphovascular space invasion (LVSI), rather than relying solely on anatomical extent. Our findings suggest that MTV—particularly when derived using reproducible and scanner/protocol independent methods—could serve as a valuable adjunct to both preoperative histopathological risk classification and MRI-based assessments. PET-derived volumetric metrics such as MTV and TLG may also capture aspects of biologic aggressiveness that parallel the features now driving FIGO 2023 staging. Although not direct surrogates for LVSI or other microscopic markers, the associations observed between MTV and disease-specific survival indicate that MTV may reflect tumor burden and infiltrative potential relevant to these prognostic domains.

These results align well with previous studies demonstrating the prognostic utility of MTV (and/or TLG) in gynecologic and other solid tumors [10, 11, 24–28]. Across all segmentation approaches, MTV and TLG outperformed SUV_max_, SUV_90p_, SUV_mean_, SUV_median_, and MRI-derived ATV in predicting aggressive disease features and survival outcomes. Collectively, these findings reinforce the growing consensus that MTV and TLG provide a more comprehensive representation of tumor burden than single-voxel PET intensity measures (e.g., SUV_max_) or anatomical tumor size metrics (e.g., ATV) and may therefore complement the prognostic focus of the FIGO 2023 staging system.

To our knowledge, this is the first study to systematically compare multiple MTV segmentation strategies in endometrial cancer while simultaneously evaluating the reproducibility, anatomical correlation, and prognostic performance of the derived MTVs. The excellent interobserver agreement observed for MTV_2.5_ and its associated PET metrics (all ICCs ≥ 0.90) underscores the robustness and practical simplicity of fixed SUV-based thresholds. This is further supported by a recent study from our group, which demonstrated that PET radiomic features derived from MTV_2.5_ masks were highly reproducible, with ICCs exceeding 0.75 for 98 out of 109 evaluated features [14]. Notably, the high level of agreement achieved by a novice reader relative to an experienced nuclear medicine specialist further highlights the accessibility and user-friendliness of fixed-threshold MTV segmentation. This finding is particularly relevant in an era of increasing clinical workload, suggesting that simple and reproducible MTV methods such as MTV_2.5_ may help support high-throughput workflows and reduce dependency on expert-level interpretation.

Although MTV_40%_ also demonstrated high interobserver agreement (all ICCs ≥ 0.78), the ICCs were consistently lower than for MTV_2.5_. Since relative thresholds masks depend directly on a single SUV_max_ voxel value, they are inherently more susceptible to variation introduced during outer tumor delineation. Even minor contouring differences between readers may shift which voxel attains the highest uptake, thereby disproportionately affecting the resulting MTV. This issue is particularly relevant in endometrial cancer, where spillover from adjacent high-uptake structures, such as the urinary bladder, may be inadvertently included in the outer contour. Fixed thresholds, which do not rely on reader-specific SUV_max_ values, appear less vulnerable to such variability.

Importantly, the present study demonstrates that MTV_30%_ most closely matches MRI-derived ATV, suggesting that this relative threshold may more accurately capture the anatomical volume of the metabolically active tumor tissue. These findings are in line with those reported by Arshad et al. in cervical cancer patients (*n* = 81) [29]. Interestingly, their SUV_max_-based thresholds of 25–30% reportedly yielded smallest volumetric differences (−4% to −19%), and strong correlations (ρ: 0.84–0.85) between MTVs and ATV (MRI assessed), and also excellent interobserver agreement for the MTV measurements (ICC: 0.90–0.95) [29]. Notably, and also in line with our findings, they found that the fixed 2.5 SUV threshold yielded excellent interobserver agreement (ICC = 0.94) and strong correlation with MRI-volume (ρ: 0.82–0.85), although it consistently produced larger MTVs—ranging from 22% to 28% larger than the MRI-volumes.

In cervical cancer, and in other solid tumors where PET/CT can be integrated into radiation therapy planning, concordance between MTV and ATV may be critical for accurate target delineation. In contrast, for endometrial cancer—where the majority of patients undergo hysterectomy—the clinical utility of MTV (and other PET-derived metrics) lies primarily in its ability to predict aggressive disease features, risk of recurrence, and poor outcomes. In this context, anatomical concordance with MRI-derived ATV may be less critical. Hence, the choice of MTV delineation method is task-dependent and should be guided by the specific clinical objective at hand.

While fixed SUV thresholds such as MTV_2.5_ are susceptible to variability in the background uptake—particularly in regions with high physiological activity like the liver—SUV_max_-based thresholds are inherently sensitive to image noise, acquisition and reconstruction settings, and scanner technology, as they rely on single-voxel intensity values. In our study, prior to ComBat harmonization, all SUV_max_-based MTVs (MTV_20–60%_) exhibited significant inter-scanner variability, whereas MTV_2.5_ remained stable across scanner types (Supplementary Table S2). Although such variability can be mitigated retrospectively through harmonization or normalization (as performed in this study), such adjustments are impractical in prospective or routine clinical settings.

Furthermore, none of the SUV_max_-based PET metrics outperformed the fixed threshold approach in predicting clinical outcomes. Taken together with the observed cross-scanner stability and high interobserver agreement, these findings suggest that a fixed 2.5 SUV-threshold may provide a more robust and clinically practical metric for prognostication in endometrial cancer. Although relative thresholds offer closer anatomical concordance with MRI-derived volumes, this advantage does not appear to translate into improved risk prediction in this setting. Importantly, the observed decline in predictive performance at higher SUV_max_ thresholds (for MTV_60%_) underscores the risk of overly restrictive segmentation criteria, which may exclude metabolically relevant tumor regions and reduce the potential prognostic utility of MTV. These insights are particularly relevant for the development of radiomics and machine learning models, where voxels defined by the MTV commonly serve as a foundational input to the models. Accordingly, the choice of MTV delineation strategy should be guided by the intended clinical application, with the fixed 2.5-SUV threshold emerging as a reproducible and clinically informative option for outcome prediction in endometrial cancer.

### Strengths and limitations

A key strength of this study is the use of a large, population-based cohort with standardized imaging protocols and comprehensive clinical follow-up. The inclusion of both experienced and novice readers allowed for a realistic assessment of interobserver variability, and the use of MRI-derived ATV as a reference standard provided anatomical comparison with that of PET-based volumetric metrics. Additionally, the application of ComBat harmonization ensured comparability across different PET/CT systems, addressing a common source of variability in multicenter/scanner studies.

However, several limitations should be acknowledged. First, the retrospective, single-center design may introduce selection bias and limit the generalizability of the findings to broader clinical settings. Moreover, the results obtained in this endometrial cancer cohort may not be directly applicable to other tumor types. Although MTV_2.5_ demonstrates excellent interobserver agreement and strong prognostic value in the present study, fixed absolute thresholds may perform less well in tumors with highly heterogeneous FDG uptake, in lesions with low FDG avidity, or in anatomical regions with high physiological background activity (e.g., liver). Numerous alternative segmentation strategies have been proposed, including contrast-oriented, gradient-based, iterative, clustering-based, and machine-learning-driven methods [30]. While these approaches may outperform simpler absolute or relative SUV-based thresholds under certain conditions, their limited availability in routine clinical software and greater technical complexity currently restrict widespread adoption.

Second, the MRI and PET images were acquired at different time points, and co-registration between modalities was not performed. Although technically feasible, accurate alignment of PET/CT and MRI tumor volumes is challenging in this cohort due to differences in the image orientation: PET/CT images were reconstructed in a ‘true’ axial orientation, whereas the CE T1w MRI sequence was acquired in an axial-oblique orientation perpendicular to the uterine long axis. Additionally, the low-dose non-contrast CT images commonly accompanying the PET images, provides limited anatomical detail, making reliable landmark-based alignment difficult. Consequently, a voxel-wise assessment of spatial overlap between MTV and ATV was not undertaken. Although bladder filling or bowel motion may have altered uterine position between examinations, the tumor morphology itself, as constrained by the uterine anatomy, is likely less affected by these positional differences. Nonetheless, a more accurate evaluation of spatial concordance could ideally be performed using an integrated PET-MR system.

Finally, although ComBat harmonization effectively reduced scanner-related variability, residual differences in image quality and resolution may still have influenced quantitative PET metrics.

## Conclusion

This study demonstrates that metabolic tumor volume derived from pretreatment [^18^F]FDG PET/CT is a reproducible and clinically informative imaging biomarker in endometrial cancer. Among the segmentation methods evaluated, the fixed threshold approach (MTV_2.5_) exhibited excellent interobserver agreement and a strong correlation with MRI derived anatomical tumor volume (ATV) derived from MRI. Notably, the relative threshold MTV_30%_ most closely approximated ATV in size, suggesting it may offer superior anatomical fidelity and volume concordance.

In terms of prognostic performance, MTV_2.5_ and relative thresholds ranging from MTV_20%_ to MTV_50%_ yielded comparable and consistent performance for predicting aggressive disease features, including lymph node metastases and advanced FIGO stage, as well as poor disease-specific survival. However, the SUV_max_-based MTVs (MTV_20%_–_60%_) exhibited higher interscanner variability and lower interreader agreement compared to MTV_2.5_, which may favor the fixed threshold method for potential implementation in prospective clinical settings.

Summarized, these findings support the integration of PET-based MTV assessment—particularly using fixed threshold methods—into future risk stratification models and clinical decision-making workflows in endometrial cancer. 

## Supplementary Information

Supplementary Material 1

Supplementary Material 2

Supplementary Material 3

## Data Availability

The data sets generated and analyzed during the current study are not publicly available but are available from the corresponding author upon reasonable request, and if in compliance with the general data protection regulation (GDPR) and patient consents.

## References

[1] Cancer, Norwegian Institute of Public Health. In Norway 2024 [Internet]. 2025 [cited 2025 Jul 9]. https://www.fhi.no/en/publ/2025/cancer-in-norway-2024/. Accessed 9 Jul 2025.

[2] Lu KH, Broaddus RR, Massachusetts Medical Society. Endometrial cancer. N Engl J Med. 2020;383:2053–64. 10.1056/NEJMra1514010. 33207095 10.1056/NEJMra1514010

[3] Reinhold C, Ueno Y, Akin EA, Bhosale PR, Dudiak KM, Jhingran A, et al. ACR appropriateness criteria® pretreatment evaluation and follow-up of endometrial cancer. J Am Coll Radiol. 2020;17:S472-86. 10.1016/j.jacr.2020.09.001. 33153558 10.1016/j.jacr.2020.09.001

[4] Forord - Nasjonalt. handlingsprogram med retningslinjer for gynekologisk kreft, 28.06.2021 [Internet]. [cited 2022 Apr 8]. https://www.helsebiblioteket.no/retningslinjer/gynekologisk-kreft/forord. Accessed 8 Apr 2022.

[5] Haldorsen IS, Salvesen HB. What is the best preoperative imaging for endometrial cancer? Curr Oncol Rep. 2016;18:25. 10.1007/s11912-016-0506-0. 26922331 10.1007/s11912-016-0506-0 PMC4769723

[6] Concin N, Matias-Guiu X, Vergote I, Cibula D, Mirza MR, Marnitz S, et al. ESGO/ESTRO/ESP guidelines for the management of patients with endometrial carcinoma. Int J Gynecol Cancer. 2021. 10.1136/ijgc-2020-002230. 10.1136/ijgc-2020-002230 33397713

[7] Nougaret S, Sala E, Lakhman Y, Sadowski E, Venkatesan AM, Rockall A, et al. Updated ESUR guidelines for endometrial cancer: integrating MRI with the 2023 FIGO staging revolution. Eur Radiol. 2025. 10.1007/s00330-025-11700-3. 40586816 10.1007/s00330-025-11700-3

[8] Nougaret S, Horta M, Sala E, Lakhman Y, Thomassin-Naggara I, Kido A, et al. Endometrial cancer MRI staging: updated guidelines of the European Society of Urogenital Radiology. Eur Radiol. 2019;29:792–805. 10.1007/s00330-018-5515-y. 29995239 10.1007/s00330-018-5515-y

[9] Bollineni VR, Ytre-Hauge S, Bollineni-Balabay O, Salvesen HB, Haldorsen IS. High diagnostic value of 18F-FDG PET/CT in endometrial cancer: systematic review and meta-analysis of the literature. J Nucl Med. 2016;57:879–85. 10.2967/jnumed.115.170597. 26823564 10.2967/jnumed.115.170597

[10] Fasmer KE, Gulati A, Dybvik JA, Ytre-Hauge S, Salvesen Ø, Trovik J, et al. Preoperative 18F-FDG PET/CT tumor markers outperform MRI-based markers for the prediction of lymph node metastases in primary endometrial cancer. Eur Radiol. 2020;30:2443–53. 10.1007/s00330-019-06622-w. 32034487 10.1007/s00330-019-06622-w PMC7160067

[11] Husby JA, Reitan BC, Biermann M, Trovik J, Bjørge L, Magnussen IJ, et al. Metabolic tumor volume on 18F-FDG PET/CT improves preoperative identification of high-risk endometrial carcinoma patients. J Nucl Med. 2015;56:1191–8. 10.2967/jnumed.115.159913. 10.2967/jnumed.115.159913 26045311

[12] Shim S-H, Kim D-Y, Lee D-Y, Lee S-W, Park J-Y, Lee JJ, et al. Metabolic tumour volume and total lesion glycolysis, measured using preoperative 18F–FDG PET/CT, predict the recurrence of endometrial cancer. BJOG: An International Journal of Obstetrics & Gynaecology. 2014;121:1097–106. 10.1111/1471-0528.12543. 24397772 10.1111/1471-0528.12543

[13] Hatt M, Krizsan AK, Rahmim A, Bradshaw TJ, Costa PF, Forgacs A, et al. Joint EANM/SNMMI guideline on radiomics in nuclear medicine. Eur J Nucl Med Mol Imaging. 2023;50:352–75. 10.1007/s00259-022-06001-6. 36326868 10.1007/s00259-022-06001-6 PMC9816255

[14] Fasmer KE, Gulati A, Lindås S, Krakstad C, Haldorsen IS. Predicting aggressive disease and poor outcome in endometrial cancer using preoperative [18F]FDG PET primary tumor radiomics. Eur J Nucl Med Mol Imaging. 2025. 10.1007/s00259-025-07335-7. 40498156 10.1007/s00259-025-07335-7 PMC12660434

[15] Boellaard R, Delgado-Bolton R, Oyen WJG, Giammarile F, Tatsch K, Eschner W, et al. FDG PET/CT: EANM procedure guidelines for tumour imaging: version 2.0. Eur J Nucl Med Mol Imaging. 2015;42:328–54. 10.1007/s00259-014-2961-x. 25452219 10.1007/s00259-014-2961-x PMC4315529

[16] Forsse D, Berg HF, Bozickovic O, Engerud H, Halle MK, Hoivik EA, et al. Maintained survival outcome after reducing lymphadenectomy rates and optimizing adjuvant treatment in endometrial cancer. Gynecol Oncol. 2021;160:396–404. 10.1016/j.ygyno.2020.12.002. 33317908 10.1016/j.ygyno.2020.12.002

[17] Berek JS, Matias-Guiu X, Creutzberg C, Fotopoulou C, Gaffney D, Kehoe S, et al. FIGO staging of endometrial cancer: 2023. Int J Gynecol Obstet. 2023;162:383–94. 10.1002/ijgo.14923. 10.1002/ijgo.14923 37337978

[18] Creasman W. Revised FIGO staging for carcinoma of the endometrium. Int J Gynaecol Obstet. 2009;105:109. 10.1016/j.ijgo.2009.02.010. 19345353 10.1016/j.ijgo.2009.02.010

[19] Gynekologisk kreft –. handlingsprogram [Internet]. Helsedirektoratet. [cited 2025 Jan 17]. https://www.helsedirektoratet.no/retningslinjer/gynekologisk-kreft--handlingsprogram. Accessed 17 Jan 2025.

[20] Hodneland E, Dybvik JA, Wagner-Larsen KS, Šoltészová V, Munthe-Kaas AZ, Fasmer KE, et al. Automated segmentation of endometrial cancer on MR images using deep learning. Sci Rep. 2021;11:179. 10.1038/s41598-020-80068-9. 33420205 10.1038/s41598-020-80068-9 PMC7794479

[21] Fasmer KE, Hodneland E, Dybvik JA, Wagner-Larsen K, Trovik J, Salvesen Ø, et al. Whole-volume tumor MRI radiomics for prognostic modeling in endometrial cancer. J Magn Reson Imaging. 2021;53:928–37. 10.1002/jmri.27444. 33200420 10.1002/jmri.27444 PMC7894560

[22] Hoivik EA, Hodneland E, Dybvik JA, Wagner-Larsen KS, Fasmer KE, Berg HF, et al. A radiogenomics application for prognostic profiling of endometrial cancer. Commun Biol. 2021;4:1363. 10.1038/s42003-021-02894-5. 34873276 10.1038/s42003-021-02894-5 PMC8648740

[23] Orlhac F, Eertink JJ, Cottereau A-S, Zijlstra JM, Thieblemont C, Meignan M, et al. A guide to ComBat harmonization of imaging biomarkers in multicenter studies. J Nucl Med. 2022;63:172–9. 10.2967/jnumed.121.262464. 34531263 10.2967/jnumed.121.262464 PMC8805779

[24] Chung HH, Kim JW, Han KH, Eo JS, Kang KW, Park N-H, et al. Prognostic value of metabolic tumor volume measured by FDG-PET/CT in patients with cervical cancer. Gynecol Oncol. 2011;120:270–4. 10.1016/j.ygyno.2010.11.002. 21109300 10.1016/j.ygyno.2010.11.002

[25] Huang W, Zhou T, Ma L, Sun H, Gong H, Wang J, et al. Standard uptake value and metabolic tumor volume of ^18^F-FDG PET/CT predict short-term outcome early in the course of chemoradiotherapy in advanced non-small cell lung cancer. Eur J Nucl Med Mol Imaging. 2011;38:1628–35. 10.1007/s00259-011-1838-5. 21617977 10.1007/s00259-011-1838-5

[26] Kim JW, Oh JS, Roh J-L, Kim JS, Choi S-H, Nam SY, et al. Prognostic significance of standardized uptake value and metabolic tumour volume on ^18^F-FDG PET/CT in oropharyngeal squamous cell carcinoma. Eur J Nucl Med Mol Imaging. 2015;42:1353–61. 10.1007/s00259-015-3051-4. 26067088 10.1007/s00259-015-3051-4

[27] Shady W, Kishore S, Gavane S, Do RK, Osborne JR, Ulaner GA, et al. Metabolic tumor volume and total lesion glycolysis on FDG-PET/CT can predict overall survival after (90)Y radioembolization of colorectal liver metastases: a comparison with SUVmax, SUVpeak, and RECIST 1.0. Eur J Radiol. 2016;85:1224–31. 10.1016/j.ejrad.2016.03.029. 27161074 10.1016/j.ejrad.2016.03.029 PMC5675072

[28] de Van Wiele C, Kruse V, Smeets P, Sathekge M, Maes A. Predictive and prognostic value of metabolic tumour volume and total lesion glycolysis in solid tumours. Eur J Nucl Med Mol Imaging. 2013;40:290–301. 10.1007/s00259-012-2280-z. 23151913 10.1007/s00259-012-2280-z

[29] Arshad MA, Gitau S, Tam H, Park W-H, Patel NH, Rockall A, et al. Optimal method for metabolic tumour volume assessment of cervical cancers with inter-observer agreement on [18F]-fluoro-deoxy-glucose positron emission tomography with computed tomography. Eur J Nucl Med Mol Imaging. 2021;48:2009–23. 10.1007/s00259-020-05136-8. 33313962 10.1007/s00259-020-05136-8 PMC8113292

