# Supplementary material for: Toward reproducible metabolic tumor volume quantification in endometrial cancer: optimizing [¹⁸F]FDG PET/CT tumor segmentation methods
Source: EJNMMI Res. 2026 May 28;16:87. doi: 10.1186/s13550-026-01412-0 (PMC13230389; doi:10.1186/s13550-026-01412-0)
Supplement: Supplementary file 1 — Supplementary Material 1 [file 13550_2026_1412_MOESM1_ESM.pdf]

Supplementary Table S1: PET/CT and MRI protocols

Pelvic MRI and whole-body [^{18}F]FDG PET/CT acquisition and reconstruction parameters. All scanners are from Siemens.

Pelvic MRI

MRI Scanner	*Sequence*	*Plane*	*TR/TE$_1$/ TE$_2$ (ms)*	*FA (Deg)*	*FOV (mm)*	*Voxel size (mm)*
1.5T Avanto	T1 VIBE	AO	7.2/2.6	20	250x250	1.6x1.3x2.0
3T Skyra	T1 DIXON	AO	5.9/2.5/3.7	9	250x250	1.4x1.4x3.0

Whole-body [^{18}F]FDG-PET/CT

PET Scanner	*Scan time/ speed*	*Corrections*	*Iteration/ subset*	*Filter*	*FOV (mm)*	*Voxel size (mm)*
Truepoint	3 min/bed	ATTN/SCAT	4/8	5 mm gauss	700	4.1x4.1x5.0
Vision	1.1 mm/s	ATTN/SCAT/ PSF/TOF	4/5	all pass	700	1.7x1.7x3.0

CT Scanner	*CareDose[a] (ref mAs)*	*CarekV[a] (kV)*	*Recon algoritm*	*Filter/ IR strength*	*FOV scan/ Recon (mm)*	*Voxel size (mm)*
Truepoint	yes (50/240)[b]	no (120)	FB	B19f	500/700	1.4x1.4x5.0
Vision	yes (25/210)[b]	yes (120)	IR	I30f/5	500/780	1.5x1.5x3.0

AO, axial oblique slice orientation; ATTN, attenuation correction; Deg, degrees; DWI, diffusion weighted imaging; FA, flip angle; FB, filtered back projection reconstruction; [^{18}F]FDG, fluorodeoxyglucose; FOV, field of view; IR, iterative reconstruction; MRI, magnetic resonance imaging; PET/CT, positron emission tomography combined with computed tomography; PSF, point spread function correction; RESOLVE, Readout Segmentation Of Long Variable Echo trains; SCAT, scatter correction; TE, time echo; TOF, time of flight correction; TR, repetition time; TSE, turbo spin echo; VIBE, volumetric interpolated breath-hold examination.

[a]Automatic tube current/voltage modulation system on Siemens CT scanners

[b]Reference mAs for low dose/diagnostic CT protocols
