# Supplementary material for: Toward reproducible metabolic tumor volume quantification in endometrial cancer: optimizing [¹⁸F]FDG PET/CT tumor segmentation methods
Source: EJNMMI Res. 2026 May 28;16:87. doi: 10.1186/s13550-026-01412-0 (PMC13230389; doi:10.1186/s13550-026-01412-0)
Supplement: Supplementary file 3 — Supplementary Material 3 [file 13550_2026_1412_MOESM3_ESM.pdf]

Supplementary Table S3: Predicting aggressive disease and disease specific survival

Median (95% CI) ComBat harmonized [18F]FDG PET-derived metrics for primary endometrial tumors from six different metabolic tumor volume (MTV) thresholds: SUV > 2.5 and SUV > 20%-60% of SUV_{max} (Reader 1). For comparison, median (95% CI) MRI-derived anatomical tumor volume (ATV) is also included (Reader 3). Predictive performance was evaluated using areas under receiver operating characteristics curves (AUC-ROCs) for lymph node metastases (LNM) and advanced stage (FIGO III-IV), and AUC of time-dependent (td) ROCs for 3-year disease specific survival (DSS).

tumor metric	*MTV threshold (SUV >)*	*median value (95% CI) n = 146*	*AUC-ROC LNM (yes vs. no) n = 88*	*AUC-ROC FIGO (III-IV vs. I-II) n = 146*	*AUC-tdROC DSS @ 3y n = 146*
MTV	2.5 SUV	20.4 (15.5, 24.4)	0.702 [0.544, 0.859]	0.765 [0.651, 0.878]	0.709 [0.544, 0.874]
	20% SUV_{max}	14.3 (12.6, 17.4)	0.699 [0.548, 0.850]	0.760 [0.648, 0.871]	0.702 [0.532, 0.871]
	30% SUV_{max}	9.9 (8, 12.1)	0.697 [0.547, 0.847]	0.760 [0.651, 0.868]	0.693 [0.529, 0.856]
	40% SUV_{max}	7.0 (5.3, 8.8)	0.684 [0.537, 0.832]	0.754 [0.648, 0.859]	0.698 [0.548, 0.848]
	50% SUV_{max}	4.1 (3.4, 5.8)	0.678 [0.532, 0.824]	0.758 [0.657, 0.860]	0.704 [0.559, 0.850]
	60% SUV_{max}	2.4 (1.8, 3.2)	0.644 [0.491, 0.796]	0.724 [0.625, 0.824]	0.707 [0.568, 0.845]
SUV_{max}	2.5 SUV	17.8 (16.0, 19.5)	0.531 [0.358, 0.704]	0.612 [0.489, 0.735]	0.494 [0.338, 0.651]
	20% SUV_{max}	17.7 (16.0, 19.6)	0.461 [0.286, 0.637]	0.616 [0.492, 0.740]	0.497 [0.337, 0.656]
	30% SUV_{max}	17.8 (16.0, 19.6)	0.461 [0.286, 0.637]	0.616 [0.492, 0.740]	0.494 [0.335, 0.653]
	40% SUV_{max}	17.7 (15.8, 19.4)	0.461 [0.286, 0.637]	0.617 [0.494, 0.740]	0.497 [0.339, 0.655]
	50% SUV_{max}	17.7 (15.8, 19.4)	0.461 [0.286, 0.637]	0.617 [0.494, 0.740]	0.497 [0.339, 0.655]
	60% SUV_{max}	17.8 (16.0, 19.6)	0.458 [0.283, 0.634]	0.616 [0.492, 0.740]	0.494 [0.335, 0.654]
SUV_{90p}	2.5 SUV	11.1 (9.8, 11.8)	0.521 [0.352, 0.690]	0.601 [0.482, 0.720]	0.464 [0.309, 0.619]
	20% SUV_{max}	11.8 (10.6, 13.2)	0.504 [0.333, 0.675]	0.598 [0.479, 0.718]	0.471 [0.318, 0.624]
	30% SUV_{max}	12.8 (11.5, 14.2)	0.501 [0.321, 0.681]	0.592 [0.467, 0.717]	0.474 [0.319, 0.629]
	40% SUV_{max}	13.7 (12.1, 14.9)	0.505 [0.319, 0.690]	0.595 [0.466, 0.723]	0.482 [0.324, 0.640]
	50% SUV_{max}	14.3 (12.6, 15.7)	0.485 [0.298, 0.671]	0.597 [0.468, 0.726]	0.485 [0.325, 0.644]
	60% SUV_{max}	15.4 (13.7, 16.3)	0.516 [0.332, 0.701]	0.598 [0.470, 0.726]	0.484 [0.324, 0.644]
SUV_{mean}	2.5 SUV	6.1 (5.7, 6.6)	0.492 [0.339, 0.645]	0.638 [0.529, 0.746]	0.480 [0.331, 0.628]
	20% SUV_{max}	7.4 (6.6, 7.8)	0.470 [0.311, 0.629]	0.626 [0.513, 0.738]	0.495 [0.341, 0.650]
	30% SUV_{max}	8.8 (8.0, 9.7)	0.481 [0.308, 0.654]	0.609 [0.489, 0.729]	0.485 [0.330, 0.640]
	40% SUV_{max}	10.2 (9.2, 11.1)	0.486 [0.309, 0.663]	0.603 [0.480, 0.726]	0.484 [0.330, 0.639]
	50% SUV_{max}	11.3 (10.5, 12.6)	0.484 [0.304, 0.663]	0.601 [0.476, 0.726]	0.489 [0.332, 0.646]
	60% SUV_{max}	12.7 (11.4, 14.1)	0.525 [0.343, 0.707]	0.605 [0.479, 0.730]	0.488 [0.331, 0.646]
SUV_{median}	2.5 SUV	5.1 (4.7, 5.6)	0.526 [0.378, 0.674]	0.668 [0.564, 0.772]	0.476 [0.336, 0.616]
	20% SUV_{max}	6.5 (6.0, 7.3)	0.461 [0.309, 0.614]	0.640 [0.531, 0.749]	0.502 [0.348, 0.656]
	30% SUV_{max}	8.3 (7.4, 9.0)	0.481 [0.313, 0.648]	0.613 [0.496, 0.730]	0.488 [0.331, 0.645]
	40% SUV_{max}	9.6 (8.9, 10.8)	0.485 [0.308, 0.661]	0.605 [0.483, 0.727]	0.489 [0.335, 0.644]
	50% SUV_{max}	10.8 (10.0, 12.2)	0.487 [0.307, 0.668]	0.598 [0.473, 0.724]	0.489 [0.333, 0.646]
	60% SUV_{max}	12.5 (11.1, 13.6)	0.527 [0.348, 0.706]	0.607 [0.482, 0.731]	0.485 [0.328, 0.643]
TLG (ml)	2.5 SUV	141 (101, 173)	0.696 [0.536, 0.856]	0.761 [0.650, 0.872]	0.694 [0.531, 0.857]
	20% SUV_{max}	116 (92, 151)	0.688 [0.530, 0.846]	0.762 [0.651, 0.873]	0.705 [0.545, 0.866]
	30% SUV_{max}	92 (72, 119)	0.687 [0.531, 0.843]	0.766 [0.658, 0.873]	0.688 [0.531, 0.845]
	40% SUV_{max}	74 (55, 97)	0.690 [0.543, 0.837]	0.768 [0.667, 0.869]	0.691 [0.542, 0.840]
	50% SUV_{max}	52 (39, 73)	0.684 [0.540, 0.829]	0.763 [0.667, 0.860]	0.680 [0.540, 0.821]
	60% SUV_{max}	33 (25, 45)	0.654 [0.503, 0.806]	0.739 [0.641, 0.837]	0.670 [0.528, 0.812]
ATV (ml)	na	9.5 (8.0, 12.3)	0.632 [0.454, 0.810]	0.715 [0.589, 0.840]	0.699 [0.534, 0.864]

Abbreviations: CI, confidence interval; [18F]FDG, fluorodeoxyglucose; MRI, magnetic resonance imaging; na, not applicable; PET, positron emission tomography; SUV, standardized uptake value; TLG, tumor lesion glycolysis; 90p, 90 percentile.
